# Human milk banking services: An umbrella review protocol

**DOI:** 10.12688/hrbopenres.14180.3

**Published:** 2025-12-10

**Authors:** Roisin O'Donovan, Aoife Long, Elaine Lehane, Helen Mulcahy, Siobhan Ward, Margaret Murphy, Susan Rogers, Gillian Weaver, Patricia Leahy-Warren

**Affiliations:** 1 University College Cork School of Nursing and Midwifery, Cork, County Cork, Ireland; 2 La Leche League International, Dublin, Ireland; 3 Western Health & Social Care Trust, Londonderry, UK; 4 Human Milk Foundation, Hertfordshire, UK

**Keywords:** Human milk banking; Umbrella Review

## Abstract

**Objective:**

To review the current state of the art of human milk banking including evidence on development and implementation of human milk banking services in middle- and high-income countries; models of delivery; and the roles of key stakeholders in supporting and sustaining human milk banking services.

**Introduction:**

Donor Human Milk (DHM) is human milk in excess of an infant's current and future needs that is donated for use by another infant. The World Health Organisation recommends that it should be available to all preterm and low birthweight infants. human milk banks (HMBs) require operational sustainability to ensure all year-round consistency and equity of DHM supply. This review will inform the co-design of an evidence framework for a national human milk banking resource network.

**Inclusion criteria:**

Systematic reviews of studies reporting on the development, implementation and sustainability of a milk banking service in middle- and high-income countries.

**Methods:**

The guidelines for umbrella reviews from the JBI Manual for Evidence Synthesis will be followed. A key word search strategy will be used to search: CINAHL (EBSCO), MEDLINE (EBSCO), PsycINFO (EBSCO), Embase (Ovid), JBI Evidence Synthesis, Epistemonikos, and the PROSPERO register. A grey literature search will be undertaken on Google scholar, BASE and the National Institute for Health and Care Excellence (NICE) website. English language and date restrictions (2000-current) will be used. Titles and abstracts and full-text articles will be independently screened by two reviewers. The reference lists of the included studies will be searched. Studies will be screened in Covidence by two independent reviewers. All reviewers will agree on the included studies. Data will be extracted and presented graphically using figures and tables. Narrative summary text will accompany the tables and figures.

This protocol has been registered on PROSPERO

## Introduction

The World Health Organisation (WHO) recommends that Donor Human Milk (DHM) should be available to all preterm and low birthweight infants. DHM is human milk in excess of an infant's current and future needs that is donated for use by another infant ( PATH, 2019b). In the absence of any or insufficient Mothers Own Milk (MOM), DHM, obtained from a human milk bank (HMB) facility, is recommended for the care of preterm or low birthweight infants ( World Health Organization, 2011; World Health Organization, 2022). When supplementation is required, DHM should be used as an alternative to formula and serve as a bridge to ensure an exclusively human milk diet through providing lactation support to build mothers milk supply ( PATH, 2019b) or, where needed, as an infant’s sole source of human milk.

Human milk is recognised as a pillar of child survival globally ( Victora *et al.*, 2016) and is the optimal diet for preterm infants ( Cleminson *et al.*, 2016). It provides a host of nutritional, immunological, neurological and physiological benefits to infants ( Israel-Ballard *et al.*, 2024; Ong & Belfort, 2021). An exclusively human milk diet during the first days of life is associated with decreased infection, morbidity, and mortality in very low birth weight infants ( Corpeleijn *et al.*, 2012; Lapidaire *et al.*, 2022; Lucas & Cole, 1990; Meinzen-Derr *et al.*, 2009; Sisk *et al.*, 2007). Preterm infants who are exclusively fed human milk are less likely to suffer from necrotising enterocolitis compared to formula fed infants ( Lucas & Cole, 1990; Quigley *et al.*, 2024; Sisk *et al.*, 2007). However, preterm and low birth weight infants often do not have access to their own mother’s milk in the first hours or days, weeks or even months of their life ( Israel-Ballard *et al.*, 2024; Tudehope, 2013). Women who deliver babies preterm can encounter a variety of challenges that lead to low rates of breastfeeding. They are often separated from their infants at birth and must learn how to establish and maintain milk production by expression. This requires support both in the hospital and after they are discharged to achieve and sustain exclusive breastfeeding ( Israel-Ballard *et al.*, 2024; Tudehope, 2013). Providing access to DHM for preterm or low birthweight infants can provide them with an optimal 100% human milk diet while their mother establishes her own milk supply.

There are currently approximately 800HMB operating globally in approximately 70 countries ( Human Milk Bank Global Map, 2019). HMBs require operational sustainability to ensure all year-round consistency and equity of DHM supply. The sustainability of HMB depends on DHM supply and demand, financial sustainability, and communications, policy and advocacy to support HMB ( Israel-Ballard *et al.*, 2024). Sustainability measures that support supply and demand for a HMB include providing lactation support for mothers to maintain a donor pool, securing engagement of local stakeholders and maintaining sound financial and business practices ( Israel-Ballard *et al.*, 2024; PATH, 2019a). Community education and engagement can raise public awareness of HMB activities and normalise human milk feeding for all infants ( Israel-Ballard *et al.*, 2024). HMB services that exist within a supportive health care system, alongside governmental support, and national advertising campaigns can support awareness and the ongoing need for donors ( Herson & Weaver, 2024).

Ensuring sustainable service provision requires equitable recruitment of donors and equitable clinical provision of donor milk. Lack of equal opportunity to donate milk and access DHM exist at national and regional levels globally ( Herson & Weaver, 2024; Israel-Ballard *et al.*, 2024). Providing payments or reimbursements to milk providers is not the norm and is illegal in a few countries, however, informal channels (i.e. the internet) mean that it is often tolerated in other countries ( Herson & Weaver, 2024; Klotz *et al.*, 2022). The absence of financial or other incentives minimises the chance of donors deliberately concealing information that may impact the quality and safety of their milk (i.e. drug, tobacco and alcohol use). Providing adequate support and accurate information to enable informed consent is vital to avoid exploitation of human milk donors and their infants ( Herson & Weaver, 2024). DHM is typically allocated based on the risk of morbidity and mortality, prioritising the smallest and sickest infants to avoid the potential health risks associated with formula use ( Israel-Ballard *et al.*, 2024).

HMB should champion breastfeeding within their community and support lactation and continued breastfeeding while collecting, processing and distributing safe and high-quality DHM ( Herson & Weaver, 2024; Israel-Ballard *et al.*, 2024). It is important that DHM and HMB are integrated with the provision of comprehensive support for and promotion of exclusive breastfeeding. This involves providing lactation support services such as peer support groups, maternal health services referrals, and expression equipment. Supporting maternal lactation and breastfeeding also includes reducing the need for supplementary nutrition through avoiding separation of mother and child, promoting skin- to-skin care, and following the United Nations International Children's Emergency Fund Baby Friendly Initiative recommendations for providing practical lactation and breastfeeding support ( Herson & Weaver, 2024). Providing this maternal lactation and breastfeeding support ensures the sustainability of DHM supply and demand ( Israel-Ballard *et al.*, 2024).

Resources for establishing and integrating HMBs are openly available online, including a toolkit to guide critical steps for establishing human milk banking as an integrated component within breastfeeding support and neonatal care ( PATH, 2025). These resources provide in-depth guidance on readiness, quality assurance, operations, auditing, training, monitoring and evaluation, and communications. In May 2024, the EU Council has adopted new rules aimed at improving the safety and quality of substances of human origin (SoHO). Building on this, the IMAGINE-HMB Project is an EU-funded initiative which aims to develop evidence-based guidelines that are aligned to the EU SoHO regulation and ensure the safety and quality of donor human milk (DHM). This project will assemble a multidisciplinary forum of experts to develop and launch a comprehensive plan to assist HMBs in complying with technical and oversight standards, making the new guidelines accessible and actionable across diverse settings. In preparation for these projects, many systematic and scoping reviews have been conducted over the past 15–20 years.

This umbrella review aims to synthesize evidence from systematic reviews in order to understand how HMB services have been developed and scaled at the national level in middle- and high-income countries. It will inform the co-design of an evidence framework cognisant of governance, quality and sustainability criteria for a proposed national milk banking resource network.

A preliminary search for existing Umbrella Reviews on milk banking services was conducted using the Epistomonikos and PROSPERO databases and none were found.

## Objective

The key objectives of this review are:

- To understand how HMBs are defined in the literature.
- To identify how HMBs have been funded, developed and scaled at the national level in middle- and high-income countries.
- To identify and compare organizational models (e.g., centralized, regionalized, decentralized, or federalized systems) used in the implementation of HMBs.
- To understand the distribution/access models used for DHM and how they are impacted by DHM supply, including available guidelines and decision making of clinical staff.
- To explore the roles of key stakeholders (e.g., government agencies, hospitals, professional associations, donors and recipients) in supporting and sustaining milk bank networks.
- To examine common facilitators and barriers to the implementation of milk banks in diverse middle- and high-income contexts.

## Research questions

The overarching research question for this review is: What is the current state of the art in the development and national-level implementation of HMB service, including operational models, implementation, and integration into healthcare systems?

The specific research questions are:

- How have HMBs been defined in the literature?
- How have HMBs networks been funded, developed and scaled at the national level in middle- and high-income countries?
- What are the organizational models (e.g., centralized, regionalized, decentralized or federalized systems) used in the implementation of milk banks?
- What distribution/access models have been used for DHM and how are they impacted by DHM supply, available guidelines and decision making of clinical staff?
- What roles do key stakeholders (e.g., government agencies, hospitals, professional associations, donors and recipients) play in supporting and sustaining milk bank networks?
- What are the facilitators and barriers to the implementation of milk banks in diverse middle- and high-income contexts?

## Methods and analysis

### Design

This review will follow the guidelines for umbrella reviews from the JBI Manual for Evidence Synthesis ( Aromataris & Munn, 2020). An umbrella review is a systematic review of existing systematic reviews ( Aromataris *et al.*, 2024). Given the large number of systematic reviews that are available, umbrella reviews are conducted to compare and contrast these published reviews ( Aromataris *et al.*, 2024). Umbrella reviews allow researchers to address a broad scope of issues related to a topic of interest- this umbrella review will provide an overall examination of a body of information available on developing and implementing a HMB in middle- or high-income countries. This will provide an exploration of the approaches taken internationally to inform the acceptability, development, implementation, and maintenance model of a national HMB.

### Inclusion criteria

Systematic reviews of studies reporting on the development and implementation of a milk banking service in middle and high income countries. Reviews published in English since 2000 will be considered for inclusion. For the purposes of an Umbrella Review, the term “studies” will refer exclusively to syntheses of research evidence including systematic reviews and meta-analyses.

***Participants***

Prospective, current or past human milk donors; donor milk recipient families; staff working within HMB service.

***Phenomena of interest***

This review is focused on HMB service and the provision of donor human milk.

***Outcomes***

The acceptability, development, implementation, and maintenance of affordable, effective, efficient, safe and sustainable HMB service.

***Context/Setting***

HMBs or HMB service operating in any middle- and high-income country.

***Types of studies***

Existing systematic reviews and meta-analyses. All types of syntheses of research evidence will be included, including systematic reviews in their various forms (effectiveness, meta-aggregative, integrative, meta-synthesis etc.) and meta-analyses.

***Exclusion criteria***

Reviews that do not report on the development or implementation of a HMB service. Reviews not available in English. Reviews that do not focus on middle- and high-income countries.

### Search strategy

A three- phase search process will be used to locate both published and unpublished reviews ( Aromataris *et al.*, 2024). An initial search was carried out on PubMed and CINAHL and Medline to identify articles on the topic. The initial keywords are informed by the Special Issue of Maternal and Child Nutrition Volume 20: Expert overview of critical focus areas towards the development of global HMB standards from July 2024.

The keywords contained in the Special Issue were used to develop a full search strategy ( Table 1 and Table 2). The search strategy was reviewed by topic experts and knowledge users who are professionals in this are.

**Table 1.** Developing the Search Strategy.

Search concepts	HMB Service	Synthesis studies
**Search terms**	“donor milk” OR “milk bank*” OR “milkbank*” OR “human milk bank*” OR “donor human milk” OR “milk donation” OR “donor breastmilk” OR “donor breast milk” OR “human donor milk” OR “human milk donor” OR “Breast* milk bank*” OR "human milk shar*" OR "milk shar*" OR "milk kinship" OR "nurs* milk" OR "cross* milk" OR "milk sibling*"	“systematic” OR “meta- analysis” OR “meta-aggregative” “integrative review” OR “meta-synthesis”

**Table 2.** Search Strategy.

Search	Query
**#1**	**Title/Abstract:** “donor milk” OR “milk bank*” OR “milkbank*” OR “human milk bank*” OR “donor human milk” OR “milk donation” OR “donor breastmilk” OR “donor breast milk” OR “human donor milk” OR “human milk donor” OR “Breast* milk bank*” OR "human milk shar*" OR "milk shar*" OR "milk kinship" OR "nurs* milk" OR "cross* milk" OR "milk sibling*"
**#2**	**Title/Abstract:** “systematic” OR “meta- analysis” OR “meta-aggregative” “integrative review” OR “meta-synthesis”
**#3**	#1 AND #2
**Limits**	2000-present

Database-specific searches will be conducted for CINAHL (EBSCO), MEDLINE (EBSCO), PsycINFO (EBSCO), PubMed, and Embase (Ovid). Major repositories of systematic reviews will also be searched, namely JBI Evidence Synthesis, Epistemonikos, and the PROSPERO register. A grey literature search will be undertaken on Google scholar, BASE and the National Institute for Health and Care Excellence (NICE) website. The first 100 items, sorted by relevance, on Google scholar will be screened. All items from other databases will be screened. The reference list of all included reviews will be searched. Date restrictions (2000-current) will be used. One full search has been included in this protocol ( Table 3).

**Table 3.** Full Sample Search String for CINAHL.

Search	Query	Records Retrieved
**#1**	**Title/Abstract:** (MH "Donor Milk") OR (MH "Milk Banks") OR “milkbank*” OR “human milk bank*” OR “donor human milk” OR “milk donat*” OR “donor breastmilk” OR “donor breast milk” OR “human donor milk” OR “human milk donor” OR “Breast* milk bank*” OR "human milk shar*" OR "milk shar*" OR "milk kinship" OR "nurs* milk" OR "cross* milk" OR "milk sibling*"	**1,222**
**#2**	**Title/Abstract:** (MM "Systematic Review") OR "systematic" OR (MM "Meta Analysis") OR "Meta- analysis" OR "meta analysis" OR “meta-aggregative” “integrative” OR “meta-synthesis”	**269,543**
**#3**	#1 AND #2	**48**
**Limits**	2000-present	

## Screening

All identified articles will be uploaded to Covidence. Duplicates will be removed. Title and abstracts of the remaining articles will be screened by two independent reviewers. The full text of all articles that meet the inclusion criteria will then be reviewed and screened by two independent reviewers on Covidence. If there are any disagreements between reviewers during the screening process, a third reviewer will assess the relevant records, and consensus will be reached on eligibility through discussion. Reasons for article exclusion will be reported. Results of the screening process will be presented in a PRISMA flow diagram ( Tricco *et al.*, 2018).

### Data extraction

Two independent reviewers will conduct data extraction. A data extraction template based on the standardised JBI data extraction tools will be used. This template will be adapted to align with the review objectives and research questions ( Table 4). It will be piloted and revised, if needed, before use. The following categories will be included in the data extraction tool; author, year, country, type of evidence source, study aim and objectives, participants, context, HMB service type, approach to HMB service development, HMB service organisational model, role of key stakeholders, implementation facilitators and barriers. Any disagreements between reviewers will be resolved through discussion or with a third reviewer. If necessary, authors of papers will be contacted to request missing or additional information.

**Table 4.** Data Extraction Instrument.

Author/ Year/Review Type	Objectives	Participants (characteristics/ total number)	Setting/ context	Search details (sources searched/ date range/ number studies included)	Study appraisal rating	HMB service type/model- including distribution/ access models	Role of key stakeholders – Governance and Personnel?	Development/ Implementation process, facilitators and barriers	Funding source/ type	Factors impacting Sustainability (including acceptability) and Maintenance of HMB	Donor Experience

### Data analysis

All extracted data will be mapped and summarised according to the aims and objectives of this review. All included systematic reviews will be summarised based on the studies’ characteristics, including the year, study aim and objectives, participants, context and HMB characteristics. Individual studies identified in each included review will be extracted and integrated to identify the number of studies that were included in more than one review. This will be reported in our findings and any potential for “double coding” will be acknowledged transparently.

Narrative summaries will outline characteristics of HMBs, including organizational models used; roles of key stakeholders; facilitators and barriers to development and implementation. This information will be analysed to build our understanding of the current state of the art in human milk banking services. Findings from reviews that included diverse income setting will be integrated narratively unless income setting is reported separately in the original review. Data will be presented graphically using figures and tables which will be accompanies by narrative summaries to explain the results presented.

We will adopt a reflexive approach throughout the review process. This will involve reviewers engaging in critical reflection throughout all the stages outlines above, namely study screening, data extraction, and interpretation of the finding. They will be conscious of the potential biases inherent in the synthesis process and will disclose their perspectives, decisions made during the review process, and how these may influence findings in the final report where relevant. These processes will be reported in a reflexive statement to provide a richer understanding of the decisions and interpretation that influenced the review findings.

### Quality assessment

The JBI critical appraisal instrument for Systematic reviews and Research Syntheses will be used to assess the quality of all included studies.

### Data summary

A narrative synthesis will be presented to summarise the range of over-arching structures, processes and approaches employed internationally to support the development and implementation of a HMB service. Analysis will be conducted in collaboration with topic experts and knowledge users who are professionals in this area. Professionals involved with HMB services in Ireland and people with lived experience of experience of both receiving DHM and donating her breastmilk will review and provide feedback on findings. The results of the review will inform the co-design of a framework for a national HMB service. Any overlaps of original research studies in each of the included research syntheses will be clearly indicated in the review findings.

## Ethics and consent

Ethics and consent were not required

## Data Availability

PRISMA-P Checklist: Available on Zenodo under CC0 license. DOI. https://doi.org/10.5281/zenodo.15624176 ( O’Donovan, 2025) No other data are associated with this article.
