# Supplementary material for: Human milk banking services: An umbrella review protocol
Source: HRB Open Res. 2025 Dec 10;8:69. Originally published 2025 Jun 19. [Version 3] doi: 10.12688/hrbopenres.14180.3 (PMC12780515; doi:10.12688/hrbopenres.14180.3)
Supplement: Peer review history

# Reviewer report: Reviewer response for version 3

**DOI:** 10.21956/hrbopenres.15749.r52184

**Published:** 2026-01-07

**Authors:** Aleksandra Purkiewicz

**Affiliations:** 1 University of Warmia and Mazury in Olsztyn, Olsztyn, Warmian-Masurian Voivodeship, Poland

The presented umbrella review covers an important global issue concerning infant nutrition and breastfeeding.

 The structure of the paper is appropriate. I have only a few minor comments, which I would like the authors to address:

 World Health Organization instead of World Health Organisation in the abstract.

 Abstract - Introduction – add the abbreviation World Health Organization in brackets – (WHO).

 In the introduction, please indicate how widespread the use of Donor Human Milk is worldwide.

 Please consider presenting a search strategy in the figure, indicating how many articles were found, how many were excluded, and how many were included in the analysis. Visualizing the figure may help the reader understand the methodology used by the authors.

Is the study design appropriate for the research question?

Yes

Is the rationale for, and objectives of, the study clearly described?

Yes

Are sufficient details of the methods provided to allow replication by others?

Yes

Are the datasets clearly presented in a useable and accessible format?

Yes

Reviewer Expertise:

human milk quality, food science

I confirm that I have read this submission and believe that I have an appropriate level of expertise to confirm that it is of an acceptable scientific standard.

# Reviewer report: Reviewer response for version 2

**DOI:** 10.21956/hrbopenres.15661.r50755

**Published:** 2025-11-22

**Authors:** Gulustan Ozturk

**Affiliations:** 1 University of Wisconsin–Madison, Madison, USA

This revised umbrella review protocol is well-conceived, timely, and highly relevant to global neonatal nutrition and maternal–child health. The manuscript demonstrates strong alignment with international priorities and fills an evident gap in systematically synthesizing evidence on human milk banking (HMB) implementation models. The revision clearly addresses earlier reviewer feedback and strengthens methodological transparency. Overall, the paper is well written and scientifically sound, with only minor recommendations for refinement.

 The revised protocol notes that reviews, including studies from low-income countries, were retained because country-level details were often unclear. The authors may wish to clarify how they plan to handle and interpret findings from diverse income settings—e.g., whether these will be reported separately or integrated narratively.

 Table 4: The data extraction criteria are thorough and well aligned with JBI guidance. However, adding a few contextual and methodological fields could enhance the depth and transparency of the final synthesis—specifically: **country or region**, **review methodology type**, and **key outcomes or recommendations**. These additions would allow more meaningful cross-context comparisons and strengthen the interpretation of facilitators, barriers, and sustainability factors.

 Since the review’s output aims to inform national resource design, a brief statement on how stakeholders (e.g., clinicians, policymakers, milk bank managers) will be engaged in interpreting findings would improve impact and transparency.

 Because umbrella reviews often include overlapping primary studies across systematic reviews, the authors should clarify how they will identify and manage overlap—for example, by constructing a citation matrix. This will ensure that evidence is not double-counted and that synthesis remains transparent.

 ​​​​Standardize terminology: use “human milk banking (HMB)” consistently throughout.

 Check table formatting and ensure search strings (Tables 1–3) are complete and reproducible.

 Minor typographical edits (spacing, capitalization of “Human Milk Bank,” etc.).

*Accept with minor revisions* — The protocol is comprehensive, well structured, and methodologically rigorous. Addressing the points above will further strengthen transparency and analytical depth.

Is the study design appropriate for the research question?

Yes

Is the rationale for, and objectives of, the study clearly described?

Yes

Are sufficient details of the methods provided to allow replication by others?

Yes

Are the datasets clearly presented in a useable and accessible format?

Yes

Reviewer Expertise:

dairy processing, dairy, bovine milk, milk bioactives, human milk, human milk procesing,

I confirm that I have read this submission and believe that I have an appropriate level of expertise to confirm that it is of an acceptable scientific standard, however I have significant reservations, as outlined above.

# Response

**Published:** 2025-12-05

**Authors:** Roisin O'Donovan

**Affiliations:** University College Cork School of Nursing and Midwifery, Cork, County Cork, Ireland

Dear reviewer,

 Thank you for taking the time to review our protocol, please see response to your helpful comments below:

- Findings from reviews that included diverse income setting will be integrated narratively unless clearly separated in the original reviews.
- Thank you for your suggested additions to our data extraction template. To clarity, 1) country or region will be included under the setting/context setting category; 2) Review methodology type will be included within the “review type” category 3) relevant outcomes or recommendations related to each category included along with results.
- We have now added a statement on how stakeholders will be engaged in interpreting findings to our data summary section.
  We have now added a statement to our data analysis section explaining how overlap in studies will be managed and reported.
- We have reviewed and updated all identified terminology and formatting issues. We use the acronym HMB to refer to a human milk bank and we use the full term “human milk banking” where relevant.

# Reviewer report: Reviewer response for version 2

**DOI:** 10.21956/hrbopenres.15661.r49426

**Published:** 2025-09-03

**Authors:** Tengfei Li

**Affiliations:** 1 University of North Carolina, Chapel Hill, USA

My comments were all addressed. I have no other concerns

Is the study design appropriate for the research question?

Yes

Is the rationale for, and objectives of, the study clearly described?

Yes

Are sufficient details of the methods provided to allow replication by others?

Yes

Are the datasets clearly presented in a useable and accessible format?

Partly

Reviewer Expertise:

Statistics; biostatistics; machine learning; imaging genetics analyses

I confirm that I have read this submission and believe that I have an appropriate level of expertise to confirm that it is of an acceptable scientific standard.

# Reviewer report: Reviewer response for version 1

**DOI:** 10.21956/hrbopenres.15595.r47944

**Published:** 2025-08-14

**Authors:** Tengfei Li

**Affiliations:** 1 University of North Carolina, Chapel Hill, USA

The authors present a well-structured umbrella-review protocol that first supplies a clear, evidence-based rationale for studying human-milk-bank services, then lays out objectives, review questions and methods that follow JBI guidance. The planned search strategy—combining controlled vocabulary, proximity operators and grey-literature limits—and the accompanying data-extraction template together form a sound, transparent framework for identifying and synthesising relevant systematic reviews. I have some suggestions:

 1) Several phrases ( *“milk sibling”*, *“milk shar*”*) may retrieve much informal sharing literature unrelated to formal HMB services; consider keeping them only if scoping showed they appear in titles of relevant reviews.

 2) specify *screening depth* (e.g. first 200 results sorted by relevance OR by year) to maintain reproducibility. 

 3) Expanding the extraction sheet with income tier and review-type fields will help organise results for the specific questions you posed. Your research questions explicitly compare middle- vs. high-income countries and will likely observe differences across review designs (systematic, scoping, qualitative meta-synthesis). Adding two columns "income tier" and "review type" to Table 4 makes it easy to sort data later.

 Author Year Country Income tier Review type… other 

 There are some minor issues:

 1)  “World Health Organzation”, or should it be “Organization”?

 2) "high-income counties" or "countries" ?

 3) “HMB require operational sustainability”, or  “HMB requires”.

Is the study design appropriate for the research question?

Yes

Is the rationale for, and objectives of, the study clearly described?

Yes

Are sufficient details of the methods provided to allow replication by others?

Yes

Are the datasets clearly presented in a useable and accessible format?

Partly

Reviewer Expertise:

Statistics; biostatistics; machine learning; imaging genetics analyses

I confirm that I have read this submission and believe that I have an appropriate level of expertise to confirm that it is of an acceptable scientific standard.

# Response

**Published:** 2025-08-26

**Authors:** Roisin O'Donovan

**Affiliations:** University College Cork School of Nursing and Midwifery, Cork, County Cork, Ireland

Thank you for your review and feedback on our Umbrella Review protocol. In response to your suggestions:

 1) We have kept our original search strategy as scoping searches found they appeared in some relevant titles.

 2) We have now specified that the depth of screening for Google Scholar was the first 100 results, sorted by relevance. All identified items were screening for the other databases.

 3) We have added “review type” to Table 4. However, once we completed screening for this review, it was clear that it would not be possible to filter reviews by country. Identified reviews included studies conducted in both high and low-and-middle-income countries or did not clearly report the countries for individual studies. Considering the goal of an Umbrella Review is to summarize or synthesize the findings of systematic reviews, and not individual studies, we did not exclude studies or findings from lower-income countries. Therefore, our results are not restricted to middle- and high income countries, as originally planned in our study protocol.

 Minor issues:

 We have now corrected each of these typos in the updated version of our protocol.
